# Specificity of Celbrea, a novel noninvasive thermal screening device, in women with recent negative screening mammograms

**DOI:** 10.3389/fonc.2026.1810073

**Published:** 2026-07-17

**Authors:** Nickolas Stabellini, Ashley Hendrix, Lisa McKnight, Linnea Cripe, Alejandra Alvarez, Jorge Salazar, Joseph H. Dayan, Martin Fleming, Alberto J. Montero

**Affiliations:** 1 Case Western Reserve University, Cleveland, OH, United States; 2 Department of Hematology-Oncology, University Hospitals Seidman Cancer Center, Cleveland, OH, United States; 3 Immunomonitoring Laboratory, Center for Immunotherapy & Precision Immuno-Oncology, Cleveland Clinic, Cleveland, OH, United States; 4 Division of Cardiology, Department of Medicine, Medical College of Georgia at Augusta University, Augusta, GA, United States; 5 Regional One Health, Memphis, TN, United States; 6 Welwaze Medical Inc, Miami, FL, United States; 7 The Institute for Advanced Reconstruction, Red Bank, NJ, United States; 8 Department of Surgery, Division of Surgical Oncology, The University of Tennessee Health Science Center, Memphis, TN, United States

**Keywords:** breast cancer, breast disease, complementary, device, mammography, screening

## Abstract

**Background:**

Breast cancer screening through mammography (MAM) remains the gold standard for early detection. However, MAM implementation faces several challenges, including psychological barriers, resource constraints, and reduced sensitivity in certain populations. Novel screening tools, complementary to mammography, may improve breast cancer detection rates and patient adherence to screening. This study evaluated whether negative results from Celbrea, a novel thermal-based device, were in agreement with negative MAM findings.

**Methods:**

We conducted a single-center retrospective cohort study at an imaging center in Memphis, Tennessee. Eligible participants were healthy women aged 25–80 years with bra cup size D or smaller, normal breast skin, and no prior breast cancer diagnosis undergoing routine annual screening MAM. Participants were screened with Celbrea, an FDA-cleared thermal device for breast disease detection. MAM results were classified as negative (BI-RADS 1 or 2) or positive. Celbrea results were classified as either: non-significant (temperature differential ≤2°F) or significant. We calculated specificity and negative predictive value (NPV) using confusion matrices.

**Results:**

Of 77 enrolled women, 68 had conclusive results for Celbrea. After excluding 5 women with BI-RADS 0 MAM, 63 were included in the final diagnostic performance analysis. Among these, 56 had negative results on both tests, 1 had a positive MAM (BI-RADS 3) with a non-significant Celbrea result, and 6 had negative MAM with significant Celbrea findings. Overall specificity was 90.3% (95% CI: 80.1-96.4) and NPV was 98.3% (95% CI: 90.6-100.0). Among women with almost entirely fatty or scattered fibroglandular tissue (n=35), specificity was 85.3% (95% CI: 68.9–95.1) and NPV was 96.7% (95% CI: 82.8–99.9). Among women with heterogeneously or extremely dense breasts (n=28), specificity was 96.4% (95% CI: 81.7–99.9) and NPV was 100.0% (95% CI: 87.2–100.0).

**Conclusions:**

Celbrea showed concordance of negative results with MAM. These findings demonstrate good discriminatory capacity and suggest potential clinical utility as an adjunctive or triage tool.

## Introduction

Breast cancer (BC) is the most commonly diagnosed malignancy among women and is the leading cause of cancer-related mortality worldwide (1). In 2022, an estimated 2,308,897 new cases and 665,684 deaths from BC were reported globally (1). Five year BC survival rate vary depending on stage at time of diagnosis and timeliness of treatment (2). In resource-poor settings, 5 year overall survival rates vary from 10% to 40%, as most women with BC are diagnosed at an advanced stage of the disease (2). By contrast, in settings where early detection and basic treatment are both available and accessible, 5-year survival rates for early localized BC can exceed 80% (2). In the US, the 5-year relative survival rate for localized BC is greater than 99% (3).

Screening is one of the primary strategies for early BC detection (4, 5). Globally, mammography (MAM) is recommended by most clinical guidelines as the gold standard screening modality for average-risk women (6). Despite its established safety and efficacy, MAM has notable limitations including patient discomfort and pain from breast compression, as well as increased anxiety and fear related to the screening process and the potential diagnosis of cancer (7, 8). These factors, along with prevalent myths and misconceptions about MAM, may reduce patient willingness to participate in routine recommended screenings, thereby decreasing adherence (7–9). Additionally, in healthcare resource-limited settings, challenges such as limited access to MAM equipment and trained personnel further hinder implementation (10). Collectively, these barriers underscore the need for complementary screening approaches to facilitate early detection which ultimately improves BC survival rates.

Celbrea is a non-invasive, thermal screening–based risk assessment tool which has been cleared by the US Food and Drug Administration (FDA) as an adjunct to routine physical examination including palpation, MAM and other established procedures for the detection of breast diseases (11). It has been validated through 18 multi-center clinical trials involving over 6,100 patients, demonstrating consistent sensitivity of 86% across tumor sizes and 99% negative predictive value (12–14). This method captures differential breast temperature through a patch that can be applied at home and may enable early cancer detection by leveraging the theory of increased biothermal activity within the tumor microenvironment (11, 15, 16). Currently, no other non-invasive, at-home approach that are FDA cleared, are available to complement standard BC screening procedures. The primary objective of this study was to evaluate the concordance between Celbrea and screening mammography in a cohort of women with negative screening MAM.

## Methods

### Study setting

The study setting was the East Campus Imaging Center of Regional One Health (Memphis, Tennessee, US) (17, 18).

### Study design

**Figure 1:**
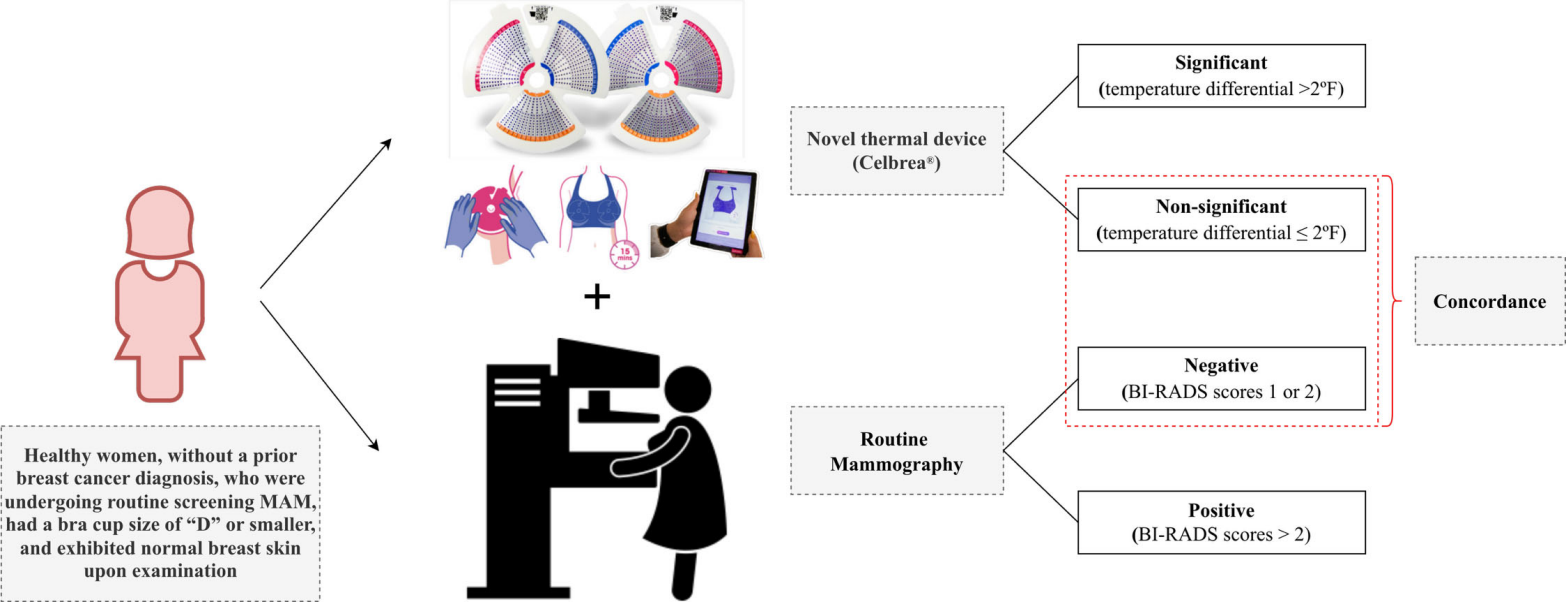
Study design overview.

A retrospective single institutional cohort study (**Figure 1**) was conducted between June 15, 2022, and May 2, 2025, including healthy women aged 25 to 80 years without a prior diagnosis of BC who were undergoing routine screening MAM, had a bra cup size of D or smaller, and exhibited normal breast skin on examination. Women were excluded if they had any of the following: breast deformities or trauma; currently lactating or breastfeeding; an active breast infection, open breast wounds, or other dermatologic conditions which would cause a temperature elevation; undergoing active chemotherapy, hormonal therapy, or radiation treatment; prior breast surgery within the past six months; indwelling wires or implants extending through the breast skin.

### Exposure

Celbrea is a novel thermal screening–based risk assessment tool that is FDA cleared for the detection of breast disease and as an adjunct to MAM (19). This device comprises two mirror-image adhesive pads, each containing three foil segments and a total of 1,188 heat-sensitive dots that undergo a colorimetric change from blue to red in response to increasing temperature. The pads are applied to both breasts for a duration of 15 minutes, after which a mobile, camera-based application automatically calculates the temperature differential. A temperature difference exceeding 2°F is considered a significant result and may indicate the presence of breast pathology.

In accordance with the product’s guidelines, the pads were applied to each participant for 15 minutes, and results were interpreted using the associated mobile application.

### Outcomes

The outcomes were the MAM result and the Celbrea result. For MAM, BI-RADS scores of 1 or 2 were considered negative, BI-RADS scores of 3–5 were considered positive, and BI-RADS 0 was considered inconclusive. BI-RADS 3 was classified as a positive MAM finding in the context of this study because the Celbrea device is intended for the detection of breast disease in general, rather than malignancy alone.

For Celbrea, results were considered negative/non-significant when the temperature differential was ≤2°F and positive/significant when the temperature differential was >2°F (20). An inconclusive Celbrea result could occur due to breast skin temperature below the detection threshold, inadequate contact between the device and the breast skin resulting from improper application, or insufficient warming of the device before application.

### Covariates

Covariates were self-reported by participants through the mobile application and included age, height, weight, body mass index (BMI), alcohol use, and tobacco history. In addition, BI-RADS score, and breast density were extracted from the MAM results.

### Statistical analysis

Categorical covariates were reported as absolute numbers and percentages. The distribution of continuous covariates was assessed using histograms and the Kolmogorov-Smirnov test. Normally distributed variables were summarized as mean and standard deviation (SD), whereas non-normally distributed variables were reported as median and interquartile range (IQR).

Confusion matrices were generated to compare Celbrea with MAM results, providing two key performance metrics: specificity and negative predictive value (NPV). Specificity refers to the percentage of individuals who test negative among those without the disease, calculated as *True Negatives/(True Negatives + False Positives)* (21). NPV indicates the likelihood that a person with a negative test result truly does not have the disease and serves as a measure of the test’s accuracy. It is calculated as *True Negatives/(True Negatives + False Negatives)* (21). Two-sided 95% confidence intervals (CIs) for both metrics were estimated using the Clopper-Pearson method (22).

All analyses were performed in R (version 4.4.3) (23).

## Results

We included 77 women (**Table 1**), with a median age of 56 years (interquartile range [IQR] 47–63). The majority were African American (66.2%), with a median BMI of 30.2 (IQR 25.9–33.5), 33.8% reported never having previously consumed alcohol, and 51.9% were never smokers. The MAM results were mostly BI-RADS 1–2 (92.0%), while scattered fibroglandular tissue was the predominant breast density category (48.1%). One patient (1.3%) had a BI-RADS 3, being categorized as MAM positive.

**Table 1:** Demographic characteristics and mammogram results for the overall cohort, patients with non-significant results from the novel thermal device, and patients with negative mammogram results.

Patient characteristics	Overall	CEL non-significant	MAM negative
	n = 77	n = 62	n = 71
Age - median (IQR)	56 (47-63)	56 (47-62)	57 (48-64)
Race/ethnicity - n (%)			
White	19 (24.7)	16 (25.8)	17 (24.0)
African American	51 (66.2)	40 (64.5)	49 (69)
Other	7 (9.1)	6 (9.7)	5 (7.0)
BMI - median (IQR)	30.2 (25.9-33.5)	30.9 (26-33.9)	30.0 (25.8-33.5)
Obesity - n (%)	38 (49.4)	35 (56.5)	34 (47.8)
Alcohol - n (%)			
Monthly	13 (16.9)	10 (16.1)	12 (17.0)
Weekly	6 (7.8)	5 (8.1)	5 (7.0)
Daily	4 (5.2)	2 (3.2)	4 (5.6)
Current (Non specified)	5 (6.5)	5 (8.1)	5 (7.0)
Former	3 (3.9)	2 (3.2)	3 (4.2)
Never	26 (33.8)	21 (33.9)	25 (35.0)
Unknown	20 (26.0)	17 (27.4)	17 (24.0)
Smoking - n (%)			
Current	15 (19.5)	9 (14.5)	14 (20.0)
Former	4 (5.2)	3 (4.8)	4 (5.6)
Never	40 (51.9)	35 (56.5)	38 (54.0)
Unknown	18 (23.4)	15 (24.2)	15 (21.0)
MAM results - n (%)			
BI-RADS 0	5 (6.5)	5 (8.1)	0
BI-RADS 1-2	71 (92.0)	56 (90.0)	71 (100.0)
BI-RADS 3-5	1 (1.3)	1 (1.6)	0
Breast density - n (%)			
Category A: Almost entirely fatty	4 (5.2)	3 (4.8)	4 (5.6)
Category B: Scattered fibroglandular tissue	37 (48.1)	29 (46.8)	34 (48.0)
Category C: Heterogeneously dense	31 (40.3)	26 (41.9)	29 (41.0)
Category D: Extremely dense	5 (6.5)	4 (6.5)	4 (5.6)

CEL, novel thermal device; MAM, mammogram; IQR, interquartile range; BMI, body mass index.

**Figure 2:**
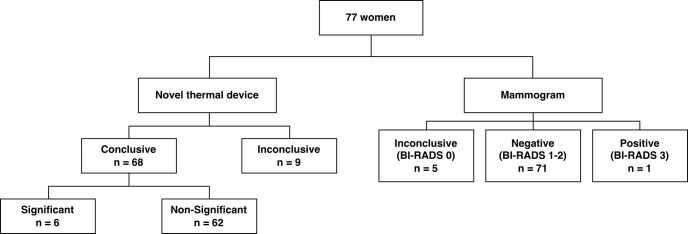
Flowchart illustrating the distribution of results from the novel thermal device and mammography within the overall cohort.

Celbrea results were non-significant in 62 individuals, while 71 had a negative MAM (**Figure 2**). Both of these subgroups showed similar demographic characteristics to the overall cohort (**Table 1**).

Excluding those with BI-RADS 0 (n = 5), and those with inconclusive results from the Celbrea device (n = 9): 56 women had a non-significant Celbrea result and a negative MAM; 1 woman had a non-significant Celbrea result and a positive MAM; and 6 had a significant Celbrea result and a negative MAM (**Table 2**). Therefore, the specificity and negative predictive value (NPV) of the Celbrea device were 90.3% (95% CI: 80.1%-96.4%) and 98.2% (95% CI: 90.6%-100.0%), respectively (**Table 3**).

**Table 2:** Demographic characteristics and mammogram results for patients with non-significant results in the thermal device and negative mammogram, patients with significant results in the thermal device and negative mammogram, and patients with non-significant results in the thermal device and positive mammogram.

Patient characteristics	CEL NS & MAM neg	CEL NS & MAM pos§	CEL SIG & MAM neg
	n = 56	n = 1	n = 6
Age - median (IQR)	57 (48 - 64)	49	57 (45 - 65)
Race/ethnicity - n (%)			
White	14 (25%)	1	0 (0%)
African American	38 (68%)	0	5 (83%)
Other	4 (7.1%)	0	1 (17%)
BMI - median (IQR)	30.6 (25.8 - 33.9)	32.6	29.3 (28.8 - 31.0)
Obesity - n (%)	31 (55.3%)	1	2 (33.3%)
Alcohol - n (%)			
Monthly	9 (16%)	1	1 (17%)
Weekly	4 (7.1%)	0	0 (0%)
Daily	2 (3.6%)	0	0 (0%)
Current (Non specified)	5 (8.9%)	0	0 (0%)
Former	2 (3.6%)	0	1 (17%)
Never	20 (36%)	0	4 (67%)
Unknown	14 (25%)	0	0 (0%)
Smoking - n (%)			
Current	8 (14%)	0	3 (50%)
Former	3 (5.4%)	0	1 (17%)
Never	33 (59%)	1	2 (33%)
Unknown	12 (21%)	0	0 (0%)
MAM Results - n (%)			
BI-RADS 0	0 (0%)	0	0 (0%)
BI-RADS 1-2	56 (100%)	0	6 (100%)
BI-RADS 3-5	0 (0%)	1	0 (0%)
Breast Density - n (%)			
Category A: Almost entirely fatty	3 (5.4%)	0	1 (17%)
Category B: Scattered fibroglandular tissue	26 (46%)	1	4 (67%)
Category C: Heterogeneously dense	24 (43%)	0	0 (0%)
Category D: Extremely dense	3 (5.4%)	0	1 (17%)

CEL, novel thermal device; MAM, mammogram; IQR, interquartile range; BMI, body mass index; NS, non-significant; SIG, significant; §Patient had a BIRADS 3, category B: scattered fibroglandular tissue.

**Table 3:** Confusion matrices, specificity, and negative predictive values comparing significant and non-significant results from the novel thermal device with positive (BI-RADS 3–4) and negative (BI-RADS 1–2) mammogram results.

Excluding BI-RADS 0				
DIagnostic tool	Result/Metric	BI-RADS 3-5	BI-RADS 1-2	TOTAL
CEL Test	Significant	0	6	6
	Non-significant	1	56	57
	TOTAL	1	62	63
	Specificity	90.32% (95% CI 80.12-96.37)		
	NPV	98.25% (95% CI 90.61-99.96)		
Breast Density A-B - excluding BI-RADS 0				
		BI-RADS 3-5	BI-RADS 1-2	TOTAL
CEL Test	Significant	0	5	5
	Non-significant	1	29	30
	TOTAL	1	34	35
	Specificity	85.29% (95% CI 68.94-95.05)		
	NPV	96.67% (95% CI 82.78-99.92)		
Breast Density C-D - excluding BI-RADS 0				
		BI-RADS 3-5	BI-RADS 1-2	TOTAL
CEL Test	Significant	0	1	1
	Non-significant	0	27	27
	TOTAL	0	28	28
	Specificity	96.43% (95% CI 81.65-99.91)		
	NPV	100.00% (95% CI 87.23-100.00)		

CEL, novel thermal device; NPV, negative predictive value; CI, confidence interval.

When stratifying these results by breast in women with category A (almost entirely fatty) or category B (scattered fibroglandular tissue) breast density, the specificity and NPV of Celbrea were 85.2% (95% CI: 68.9%-95.1%) and 96.6% (95% CI: 82.8%-99.9%), respectively (**Table 3**). In women with category C (heterogeneously dense) or category D (extremely dense) breast density, Celbrea still performed well with a specificity and NPV of 96.4% (95% CI: 81.7%-99.9%) and 100.0% (95% CI: 87.2%-100.0%), respectively (**Table 3**).

No adverse events related to the use of the device were reported.

## Discussion

The primary objective of this study was to evaluate the concordance between negative results obtained from Celbrea and a negative screening MAM, using specificity and NPV as key metrics. A total of 77 women were included, of whom 63 had conclusive results from both MAM and the thermal device. The Celbrea device demonstrated a specificity greater than 85% and an NPV exceeding 96% across the overall cohort, and across different breast density profiles, underscoring the consistency of its results with those of MAM. These findings reinforce the concordance of negative Celbrea results with MAM and support its potential application in clinical practice as an adjunctive or triage tool, particularly in settings with limited access to MAM or where resistance to screening MAM is higher. This is reinforced by recent findings from a Public Health Initiative in Northeast Argentina, which demonstrated that baseline BC risk assessment using Celbrea led to a significant increase in MAM screening rates (24).

In addition to the primary findings, our study population included a substantial proportion of women with elevated BMI and diverse racial and ethnic backgrounds, factors often linked to disparities in screening access and diagnostic accuracy (25–28). The predominance of high BMI may impact both thermal differentials and the effectiveness of traditional imaging modalities, underscoring the potential value of a non-invasive physiological assessment tool (29). Beyond its diagnostic capabilities, the device also demonstrated a favorable safety profile, with no adverse events reported during or after use. These results support its potential for broader implementation, particularly in community-based or at-home screening settings.

This novel thermal device operates on the principle that tumors exhibit increased angiogenesis and metabolic activity, leading to a higher local temperature in the tumor microenvironment compared to the surrounding healthy tissue (30–34). Angiogenesis plays a key role in both local tumor growth and the development of lymph node and distant metastases in BC (35, 36). Lawson and colleagues demonstrated that the difference in breast skin temperature among women with unilateral BC averaged 2.27°F (ranging from 1.3°F to 3.5°F), compared to the mirror area of the contralateral breast (33). Further clinical studies have suggested that the local skin temperature over a breast tumor can be at least 1.8°F higher than over healthy breast tissue (37, 38). Therefore, this novel thermal device is based on the hypothesis that this differential in biothermal activity can be measured and mapped to aid in the screening of breast abnormalities.

Evidence also demonstrates that, in addition to malignancies, certain benign breast diseases and other pathophysiological conditions can generate detectable thermal gradients on the breast skin surface (39, 40). These thermal variations may arise from localized inflammatory processes and/or vasodilation associated with increased blood flow and vascular activity (40, 41). Furthermore, studies suggest that measurable thermal differences may be present even in very small tumors and regardless of breast tissue composition or density (42–44). Although obesity and increased adipose tissue may influence absolute skin surface temperature through thermoregulatory and insulating effects, these factors do not necessarily eliminate localized asymmetric thermal patterns. Because Celbrea evaluates relative bilateral thermal differences rather than isolated absolute temperature values, clinically relevant thermal asymmetries may remain detectable even in patients with elevated BMI, who comprised a substantial proportion of the analyzed cohort.

This concept of elevated biothermal activity and this novel thermal device’s ability to detect it has been further supported by clinical data. In a study involving 2,805 women who underwent assessment with the device, 86% showed breast temperature differentials of less than 2°F between breasts (12–14). This same group also had normal clinical evaluations, with no suspicious findings on physician breast examination or MAM, conducted independently and blinded to the thermal results (12–14). Additionally, among the 2,805 participants, 15 were diagnosed with biopsy-confirmed malignancies, of which 13 had a breast temperature differential of 2°F or greater, reinforcing the hypothesized link between elevated thermal asymmetry and malignant pathology (12–14).

In the analyzed cohort, six patients demonstrated a positive Celbrea result despite having a negative MAM. Evidence suggests that the estimated lifetime probability of receiving at least one false-positive result is approximately 85.5% (± 0.9%) among individuals adhering to United States Preventive Services Task Force (USPSTF) screening recommendations (45). False-positive results are an inherent limitation of screening modalities and should be considered in the context of overall screening performance (46). The primary objective of screening tools is to balance sensitivity and specificity in order to reduce unnecessary downstream testing and the associated psychological, medical, and financial consequences (45–47). In this study, the observed false-positive rate of 9.5% should be interpreted in the context of Celbrea’s intended and FDA approved use as an adjunct tool rather than as a standalone diagnostic or screening modality. Within a sequential screening pathway, some degree of false-positive results is expected and reflects the trade-off inherent to earlier-stage risk stratification approaches.

From a clinical perspective, these findings are especially relevant in settings where MAM is limited or underused due to economic, geographic, or cultural barriers. In such contexts, a tool with high NPV may aid in efficiently triaging patients and determining the need for further evaluation. Its user-friendly design, portability, and potential for self-administration make it well-suited for expanding access to early detection, particularly among underserved and hard-to-reach populations. The strong alignment with negative imaging results reinforces its value as a complementary approach to breast health monitoring, especially in younger or higher-risk groups where traditional screening may be less effective or accessible. However, caution should be exercised when interpreting NPV alone, given the low number of patients with positive MAM findings in this study (48).

### Limitations

This study has several limitations. The use of data from a single center may introduce selection bias, limiting the generalizability of the findings to the broader population. The relatively small sample size, particularly after excluding patients with inconclusive Celbrea results and BI-RADS 0 mammograms, reduces statistical power and may hinder external validation. Additionally, the limited number and scope of available covariates may affect the ability to fully account for potential confounders. Some covariates were self-reported, which introduces the possibility of misreporting or recall bias. One major limitation is the low number of positive MAM cases and biopsy-confirmed malignancies, along with the lack of longitudinal follow-up, which restricted the assessment of sensitivity, false-negative rates, and long-term clinical outcomes. The high NPV observed may have been influenced by the low prevalence of the outcome, as reflected by the small number of patients with positive MAM findings. Although the mobile application automates result interpretation, variability in device application or environmental factors may still influence skin temperature measurements.

## Conclusions

In conclusion, Celbrea demonstrated good concordance with negative MAM results, as reflected by its specificity and NPV. Its ease of use and potential for at-home application make it a promising complementary approach to breast health monitoring. It may be especially valuable in settings with limited access to MAM, higher resistance to traditional screening, and populations where conventional methods are less effective or accessible. Future studies should aim to expand validation through prospective, multicentric studies and include demographically diverse populations, with larger numbers of positive cases and a broader range of covariates.

## Data Availability

The raw data supporting the conclusions of this article will be made available by the authors, without undue reservation.
